# Painful Flexible Flatfoot Deformity: A Description of Posterolateral Ankle Trigger Point for Pain

**DOI:** 10.7759/cureus.76870

**Published:** 2025-01-03

**Authors:** Farid Kassab, Ahmed M Sonbol

**Affiliations:** 1 Orthopedic Surgery, Andalusia Hospital, Jeddah, SAU; 2 Orthopedic Surgery, Dr. Soliman Fakeeh Hospital, Jeddah, SAU

**Keywords:** flatfoot in children, flexible flatfoot deformity, foot examination, pediatric foot pain, pes planus

## Abstract

Background

Flexible flatfoot deformity may be painful, and it is often difficult to pinpoint one painful area. Considerable variability of symptoms and areas of pain are noted in this condition. This study aimed to identify a reproducible area of pain and tenderness in these types of feet, implying a painful flatfoot.

Methodology

This study included 35 patients, comprising 23 males and 12 females, with 66 painful flexible flatfoot deformities representing the study group. The average age was 7.8 years (range = 2-15). In total, 31 patients had bilateral pain, and four patients had unilateral pain. The control group included 20 age-matched patients with normal painless feet.

Results

In 28 patients, pain was non-localized in the lower extremity. Overall, seven patients had non-localized pain in the foot and could not identify a point of maximal tenderness. A new trigger point for pain was identified in all patients, 1.5-2 cm below and 1.5-2 cm posterior to the tip of the lateral malleolus, well behind the peroneal tendons. No pain was reported by the control group.

Conclusions

A new constant trigger point for pain was identified in all patients with painful flatfoot deformity. It is hypothesized to be due to the overloading of the posterolateral corner of the talocalcaneal joint or impingement on the calcaneofibular ligament secondary to the heel valgus. We believe that the presence of this trigger point will help clinicians confirm flatfoot deformity as the source of pain, adding another tool in the evaluation and diagnosis of this condition.

## Introduction

Children and teenagers may suffer from many conditions that lead to pain in the lower extremities. These disorders range from benign conditions such as growing pains and transient and reactive synovitis to more serious diseases such as juvenile rheumatoid arthritis. Urgent conditions include septic arthritis, which requires immediate attention, in addition to life-threatening conditions such as leukemia [1,2]. One of the recurrent themes is a normal clinical examination of a child with recurrent episodes of non-localized lower extremity pain. In this situation, due to the absence of clinical signs, the differential diagnosis may include growing pain or overuse pain. These diagnoses are retained until other pathologies cannot be identified. One of the main concerns for physicians is relying on such diagnoses only to discover an insidious underlying disease later. This situation is compounded by the fact that some of the serious diseases such as leukemia can present similarly. It is not uncommon to find in the history of a child with leukemia that the pain had been present for several months, mild to moderate in nature, recurrent with a normal clinical examination on repeated clinic visits [3-6].

Flatfoot deformity is described as heel valgus and flattening of the medial arch accompanied by pronation and abduction of the forefoot. The condition is associated with a prominence of the talar head medially, uncovering of the talonavicular joint, and tightness of the Achilles tendon. Ankle valgus can also be present and further contribute to the deformity and pain [7-9].

Flexible flatfoot deformity may be painful. Orthopedic surgeons as well as foot and ankle specialists encounter several of these cases in their offices. The presentation often includes painful feet upon exercise or walking. When the patient is asked to point to the area of maximal tenderness, it is often difficult to pinpoint one area. Usually, multiple areas on the foot are pointed to. Marked variability of symptoms and areas of pain are noted [10-15]. Identifying a positive clinical sign such as a trigger point for pain or a limitation of the range of motion of a joint can help physicians make a positive diagnosis more assuredly.

In this study, we describe children who were complaining of non-localized pain in the lower extremities and tiredness after walking with unremarkable clinical examination and blood tests. The only findings were the presence of flexible flatfoot deformity and generalized ligamentous hyperlaxity.

Although flexible flatfoot deformity is known to be occasionally painful, the problem, as described in this study, was that the trigger points for pain were not where they were expected, as described by the literature [11,12,14-16]. Because of the absence of a confirmed diagnosis, many of these children underwent follow-up appointments to ensure that a more serious problem was not being missed.

A systematic method for review is needed, with a reproducible area of pain and tenderness in these types of feet, which will imply a painful flatfoot. We have been able to find a reproducible trigger point as a predictor of symptomatic flatfoot. We propose that this will aid practitioners in confirming the diagnosis of symptomatic flatfoot deformity.

Many children seen in our department with lower extremity pain and early tiredness on walking had ill-defined pain with unremarkable clinical examination and laboratory investigations in the form of complete blood count, erythrocyte sedimentation rate, C-reactive protein, and antistreptolysin O titer. The only positive findings in these children were flexible flatfoot deformity and generalized ligamentous hyperlaxity.

At first, the feet were not considered to be the reason for their pain because the classical trigger points for pain were absent. Because of the lack of a positive diagnosis explaining the symptoms, follow-up visits were conducted for reassessment. Growing pain and overuse pain were diagnosed with reservation. Later, it was discovered that the flatfoot deformity was responsible for their pain and could explain early tiredness. The diagnosis was missed because the location of the trigger point for pain was different from that described in the literature. After confirming our findings in some of our early patients, we conducted a prospective case-control study to confirm the presence of this new painful trigger point in all our patients.

## Materials and methods

The Institutional Review Board of the International Medical Center approved our study (approval number: 2024-03-237). All our patients’ parents consented and agreed for their children to participate in the study.

**Figure 1:**
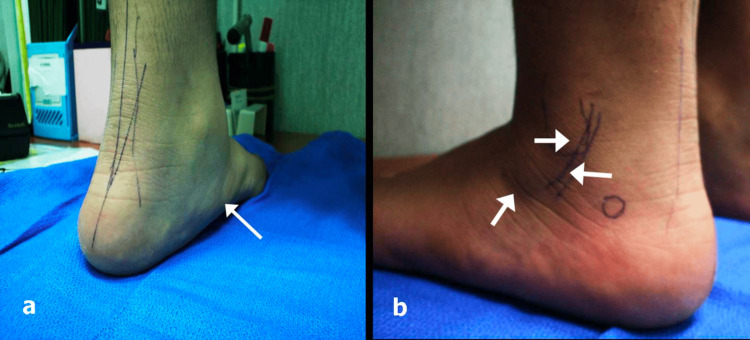
A 10-year-old boy with arrows indicating the classical trigger points for painful flatfoot deformity. (a): On the medial side: the head of the talus and the insertion of the tibialis posterior. (b): On the lateral side: the sinus tarsi, lateral malleolus, and peroneal tendons. The circle marks the newly described trigger point for pain.

**Figure 2:**
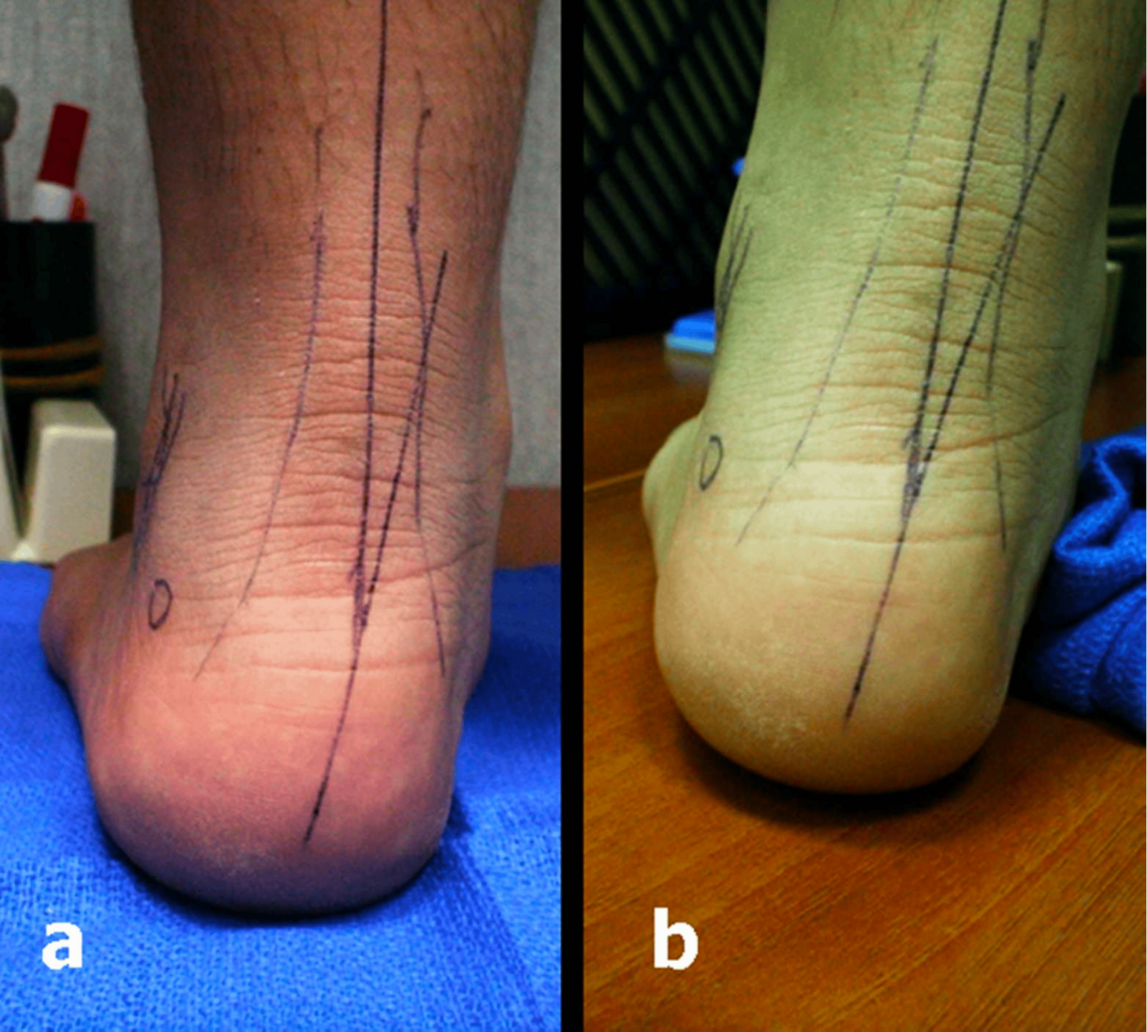
The medial arch support test in a 10-year-old boy. (a) Before inserting the foam arch support. (b) After inserting the foam arch support. Note the reduction in the heel valgus position.

This prospective study recruited 35 patients who presented to our pediatric orthopedic clinic in the International Medical Center Hospital, Jeddah, Saudi Arabia with pain in the lower extremities or tiredness after walking. The only positive physical findings were flexible flatfoot deformity and generalized ligamentous hyperlaxity. The neurological assessment was normal. The study group comprised 23 males and 12 females. Overall, 31 patients had pain in both feet, and four patients had pain in the left foot. A total of 66 painful feet were studied. The average age was 7.8 years (range = 2-15). The trigger point for pain was localized while the child was standing. After localizing the new trigger point for pain and to ensure its direct relationship to the flatfoot deformity, the children were asked to stand on a foam medial arch support for 10 minutes to correct the deformity and reduce the excessive heel valgus position (Figures 1, 2). For the non-compliant young children, the arch supports were taped to their feet, leaving the site of the trigger point uncovered. They were allowed to move freely on it for 10 minutes, following which the trigger point for pain was reassessed. Accurate assessment of pain severity, before and after correction of the deformity, using the Visual Analog Scale (VAS) was only possible in children aged 8 to 15 years (n = 15, 43%). The trigger points were then marked with a small coin, and weight-bearing lateral view foot and ankle X-rays were obtained to determine their exact anatomical location.

Patients older than 18 years of age; those with an abnormal neurological examination; those with rigid flatfoot deformity due to tarsal coalitions, congenital vertical talus, or peroneal spastic flatfoot; and those with a history of trauma were excluded.

A group of 20 age-matched children was chosen as the control group. These children presented to the unit for unrelated problems that did not involve the lower extremities with normal painless feet. The trigger point for pain was also examined in this group.

In the statistical analysis conducted in the study, a significance level of 0.05 was utilized to determine statistical significance. Simple descriptive tests were performed, and the means obtained from the VAS score were compared using a paired t-test.

## Results

The pain in our study group had a particular characteristic. In total, 28 (80%) patients were unable to identify the origin or the exact location of their pain. Vague complaints included diffuse leg or lower extremity pain. Some parents mentioned that their children were falling behind or sitting or asking to be lifted without a particular complaint. Only seven (20%) patients were able to localize their pain to the foot area. However, these patients were unable to pinpoint the exact location of their foot pain.

**Figure 3:**
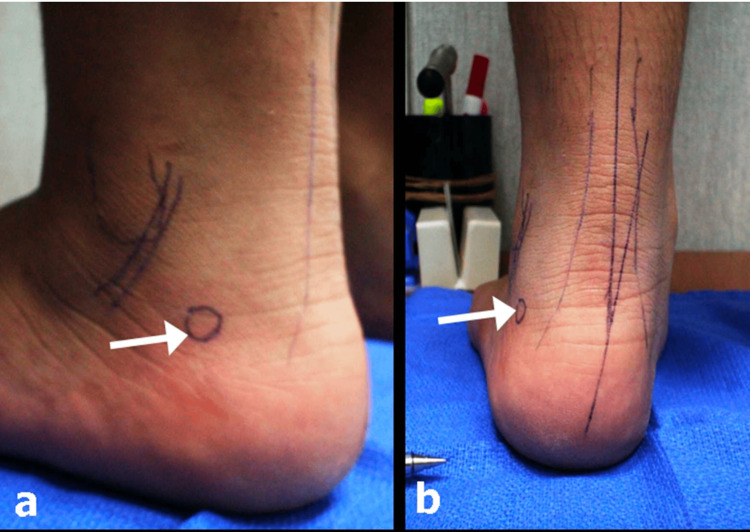
Location of the newly discovered trigger point in a 10-year-old boy for pain on the posterolateral heel, 2 cm distal and 2 cm posterior to the tip of the lateral malleolus. (a) Side view showing the position of the lateral malleolus, peroneal tendons, and tendo Achilles. (b) Back view showing an increased heel valgus position.

In all 35 patients with 66 painful feet, a trigger point for pain could be found at the posterolateral part of the heel, 1.5-2 cm inferior and 1.5-2 cm posterior to the tip of the lateral malleolus, depending on the foot size (Figure 3). Most of the pain was triggered when pressure was applied to that point in the standing position. Patients, especially young children, would jerk their feet away because of the pain.

When the same trigger point test was performed 10 minutes after insertion of the foam arch support, the pain decreased in all cases. The mean VAS score significantly decreased from 8.6 (range = 7-9) to 2.4 (range = 1-4) (p = 0.01). On the contrary, no pain was triggered in any of the children in the control group (n = 20) on pressing the same point in the standing position. The control group experienced no pain at the same point.

**Figure 4:**
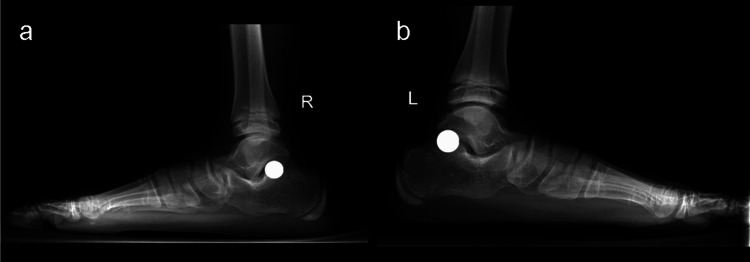
Weight-bearing lateral view plain X-rays of the right (a) and left (b) feet of the same patient in Figure 3 with painful flexible flatfoot deformity. The circular marks coincide with the position of the trigger point for pain.

X-rays of the marked feet showed the trigger point to always lie over the posterolateral part of the talocalcaneal joint (Figure 4).

## Discussion

Flexible flatfoot deformity can be painful. As described in the literature, the trigger points for pain are located medially and laterally (Figure 1). The medial pain is due to subcutaneous tissue and skin irritation and inflammation caused by the prominent head of talus or tibialis posterior tendinitis due to medial arch collapse and tendon stretching. The lateral pain is attributed to impingement in the area of the sinus tarsi, peroneal tendons, and/or lateral malleolus [11,12,14-16]. The trigger point for pain described in this study has not yet been described in the literature.

In our study group, two aspects are worth noticing. First, the majority of the patients (n = 28, 80%) were unable to localize the foot as the origin of their pain. Second, on physical examination, the tenderness point was always lateral and not medial and always at one particular point, the posterolateral corner of the calcaneus well behind the lateral malleolus and the peroneal tendons.

The fact that the medial arch support test reduced the pain led us to think that this pain was due to increased heel valgus. This is consistent with previous literature [17,18]. The excessively valgus heel appears to cause overloading of the posterolateral part of the talocalcaneal joint secondary to asymmetrical load distribution over this joint. Another possible mechanism by which excessive heel valgus likely initiates pain is the impingement of a sensitive structure between the calcaneus and talus. This appears to be different from the impingement on the peroneal tendons, the sinus tarsi, or the tip of the lateral malleolus as its location is more posterior [19,20]. These areas of impingent were free of tenderness in our study group. As the calcaneofibular ligament is the only structure crossing over the posterolateral part of the talocalcaneal joint [21], impingement on this ligament can be another theory for the pathogenesis of the new trigger point for pain. These two theories were supported by the fact that the trigger point for pain always overlaid the posterolateral part of the talocalcaneal joint, as confirmed by X-rays (Figure 4).

Before identifying this trigger point for pain in these patients, it was difficult to confirm that the pain was due to the flatfoot deformity. Given a history of non-localized pain, a normal clinical examination with the supposed trigger points for pain missing, and normal laboratory investigations, it was difficult to make a final diagnosis. We believe that identifying a specific trigger point for pain in these patients with flexible flatfoot deformity is a reassuring sign for physicians to confirm that pain is due to the flatfoot deformity and not some insidious condition.

The limitations of this study include its cross-sectional nature, which restricted the ability to establish causality. To address these limitations, future research should incorporate cadaveric, biomechanical, and magnetic resonance imaging studies to provide a more comprehensive understanding of the underlying mechanisms. Additionally, a larger study population would enhance the generalizability and robustness of the findings.

## Conclusions

The findings of the current study establish a connection between the newly identified trigger point for pain and flatfoot deformity. Although the precise pathogenesis of pain at this trigger point remains unclear, it appears to be closely linked to heel valgus deformity. The manifestation of this tenderness may be linked to the heightened stress on the posterolateral region of the talocalcaneal joint or potentially to impingement that affects the calcaneofibular ligament positioned between the talus and calcaneus. Consequently, additional evaluation in the future is warranted.

The identification of this trigger point for pain presents a valuable addition to the diagnostic repertoire for cases involving painful flexible flatfoot deformities, especially in instances where the typical points of tenderness are not evident. Moving forward, further research is essential to deepen our understanding of the mechanisms underlying pain generation at this specific trigger point and to explore its diagnostic and therapeutic implications in clinical practice.
